# Sandwiched Strut Allografts with Stem Retention to Treat Fragile Periprosthetic Femoral Fractures: A Case Report

**DOI:** 10.3390/medicina61010166

**Published:** 2025-01-20

**Authors:** Hyoung Tae Kim, Hyun Jun Lee, Suenghwan Jo

**Affiliations:** 1 Department of Orthopaedic Surgery, Chosun University Hospital, 365 Pilmundae-ro, Dong-gu, Gwangju 61453, Republic of Korea; kht2769@naver.com; 2 Department of Orthopaedic Surgery, Ulsan University Hospital, 32 Daehakbyeongwon-ro, Dong-gu, Ulsan 44033, Republic of Korea; dtscream@naver.com

**Keywords:** cable fixation, dual strut allografts, osteoporosis, periprosthetic femoral fracture, revision surgery

## Abstract

Managing periprosthetic femoral fractures is challenging, particularly in osteoporotic patients with fragile bones. Revision with a long stem is commonly considered but may fail to provide adequate fixation and stability in fragile bones. A novel approach using sandwiched strut allografts and controlled bone crushing with robust cable fixation can offer mechanical support and provide secondary stability to the loosened femoral stem and can be considered a treatment option for low-demand patients. A 73-year-old female with 23 years of hemodialysis experienced pain and instability in her right thigh following a slip. She had extremely low bone mineral density, and radiographs revealed a periprosthetic femoral fracture with a loosened femoral prosthesis, classified as Vancouver type B3. The patient underwent surgical fixation using a long anatomical plate augmented with dual strut allografts sandwiched anterior and posterior femur. Robust cable fixation was performed to partially crush the native cortical bone against the stem to enhance stability. Postoperative imaging at 18 months confirmed successful bone union and implant stability, and the patient regained preoperative functional capacity without pain. This case demonstrates that partially crushing native bone with dual strut allografts may provide stability to the loosened femoral stem and can be an effective alternative to long-stem revision surgery for patients with highly fragile bones. This approach may provide immediate mechanical stability and can be a potential treatment option for managing fragile periprosthetic femoral fractures.

## 1. Introduction

Periprosthetic femoral fractures present significant challenges in orthopedic surgery, particularly in patients with severe osteoporosis. Osteoporosis reduces bone strength, which increases the risk of re-fracture and delays the healing process. Patients undergoing chronic dialysis are especially prone to significant bone mineral loss, further diminishing bone quality. For these patients, traditional long-stem revision surgery may not be a suitable treatment [1,2,3]. Traditional treatment with long-stem revision surgery has several limitations in these patients. The stem may not be adequately fixed, and the surrounding bone may be too weak, which may ultimately result in additional fractures. This delays recovery and increases the risk of postoperative complications including mortality. Therefore, alternative surgical methods are urgently needed for patients with severe osteoporosis [4,5].

Recently, a novel approach involving the use of dual strut allografts and robust cable fixation on either side of the femur has gained attention [6]. This method enhances mechanical support and promotes bone healing, potentially offering better outcomes than traditional long-stem revision surgery. The allografts provide a scaffold for new bone growth, while the cable fixation offers immediate mechanical stability. This integrated approach is particularly promising for high-risk osteoporotic patients undergoing chronic dialysis [7].

This case report demonstrates how sandwiched dual strut allografts and partially crushing the native bone against the femoral stem can be an effective treatment option for high-risk osteoporotic patients for whom long-stem revision surgery is not a viable option [8,9].

## 2. Case Presentation

**Figure 1:**
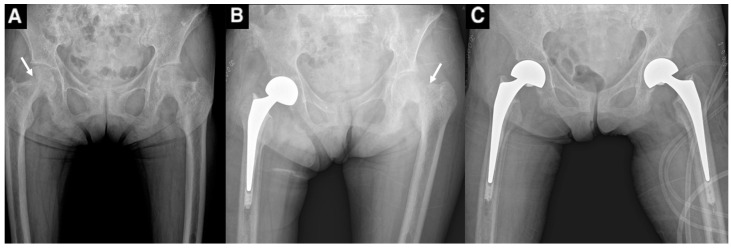
(**A**) An anteroposterior radiograph of the pelvis showing osteoporotic bone with a spontaneous femoral neck fracture on the right side (narrow arrow). (**B**) Cemented hemiarthroplasty was performed, but the patient developed a spontaneous fracture on the contralateral side after 7 days (narrow arrow). (**C**) A resultant X-ray showing cemented hemiarthroplasty on both sides.

A 73-year-old female presented to our institution with severe right thigh pain after a slip. The patient had a history of chronic dialysis, which started 23 years ago, and was subsequently diagnosed with severe osteoporosis, which was treated with ibandronate for 7 years. One year prior to presentation, she had a spontaneous femoral fracture on the right side, which was treated with a cemented hemiarthroplasty at another hospital, and an additional fracture on the contralateral side one week later, which was also treated with cemented hemiarthroplasty (Figure 1).

**Figure 2:**
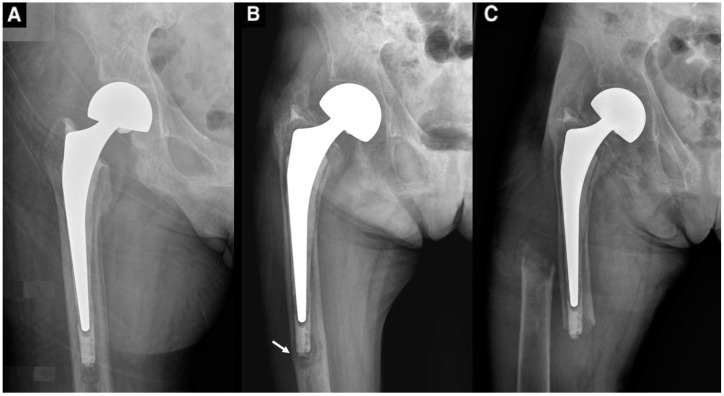
Serial X-rays showing the progression of femoral stem loosening. (**A**) An X-ray immediately following arthroplasty shows inadequate bone-to-cement fixation. (**B**) The progressive loosening of the implant 9 months following the index surgery, with impending fracture (narrow arrow), and (**C**) the resultant periprosthetic fracture at the tip of the cement 12 months following arthroplasty. Notice the thinning of the cortex over time.

Immediate postoperative X-rays showed that the cement was not fully in contact with the native femur. The operation record documented massive bleeding from the medullary canal, which may have been the reason. The patient was able to walk only with assistive devices and had limitations walking outdoors and in the community. Imaging at 9 months following the surgery revealed implant loosening on the right side. This structural instability led to bony resorption, which subsequently resulted in a periprosthetic femoral fracture after a minor fall (Figure 2).

In order to plan for the surgery, the fracture was classified using Vancouver classification. As the implant was loose and the fracture occurred at the tip of the prosthesis, this was initially classified as Vancouver type B2. However, due to the reduced bone quality and fragile bone around the fracture, it was decided that Vancouver type B3 was more appropriate. The standard treatment for Vancouver type B3 fractures typically involves revision surgery using a long stem combined with structural allografts or the use of proximal femoral allografts [6,7]. However, the patient’s femur was anterolaterally bowed, which made it difficult to use a long revision stem. In addition, due to the low bone quality, adequate stability seemed unachievable even with a long stem. Therefore, a plan was made to conduct the osteosynthesis surgery first and deal with the loosened implant afterward.

**Figure 3:**
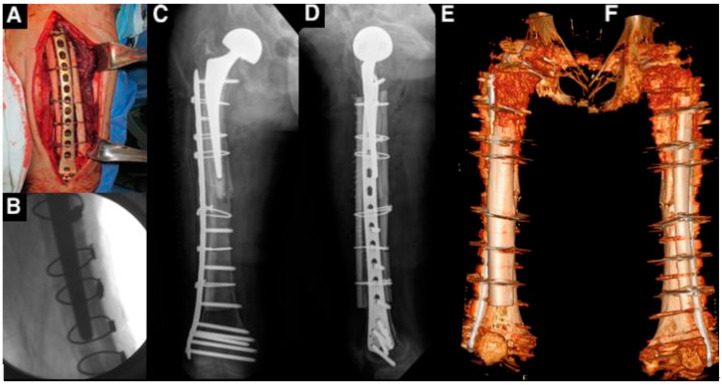
Periprosthetic fracture fixation. (**A**,**B**) A long anatomical plate was used with long strut allografts applied to the anterior and posterior sides of the femur. (**C**,**D**) The anteroposterior and lateral views of the femur, (**E**,**F**) a 3-dimensional reconstruction of the surgery.

The surgery was performed under general anesthesia. The fracture was approached, and a long anatomical plate was utilized to stabilize the fracture. For augmentation, long structural allografts were prepared and thawed at room temperature. The allografts were axially splatted with the consideration of convexity so that the convex side could be applied on the anterior femur and the concave side on the posterior [1]. The allografts and femur were firmly fixed with multiple cables, ensuring precise contact between the allografts and the native bone. After confirming the contact, the cables were additionally tensioned to create the partial crushing of the native femur against the cemented stem under fluoroscopy guidance. This meticulous fixation resulted in the secondary stabilization of the cemented stem surrounded by native bone, strut allografts anteriorly and posteriorly, and the plate [4] (Figure 3).

For postoperative care, analgesics were administered as needed. Under the guidance of a specialized physiotherapist, non-weight-bearing exercises were performed to maintain and recover muscle strength immediately. The patient was allowed to perform non-weight-bearing exercises and progressively started partial weight-bearing exercises when the pain was tolerable. The patient was able to regain preoperative ambulatory status at 4 weeks following the surgery.

**Figure 4:**
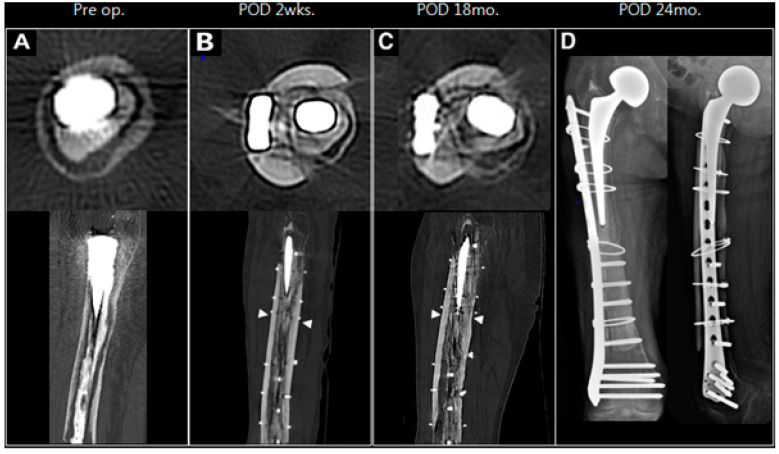
The axial and sagittal section of the femur showing the secondary stability of the cemented stem. (**A**) The preoperative CT scan shows a gap between the cortical bone and the cement. (**B**) Note the thick strut allografts placed anterior and posterior to the fractured femur, providing stability to the intramedullary cement (arrowhead). (**C**) Bone integration is confirmed, and stem stability was maintained after 18 months. (**D**) The anteroposterior and lateral views of the femur at 24 months following the operation show the unchanged position of the femoral stem as compared to the immediate postoperative status.

Follow-up assessments at 18 months post-surgery revealed that the patient maintained a pain-free range of motion in the joint. X-rays and Computed Tomography (CT) scans confirmed successful integration between the grafted allograft and the native bone and the maintained stability of the cemented stem (Figure 4).

## 3. Discussion

Treating periprosthetic femoral fractures in osteoporotic patients after hip arthroplasty is complex and challenging [3]. Patients with periprosthetic fractures are often old and have accompanied comorbidities that limit the option for surgery.

Revision surgery using a traditional long stem presents several limitations, particularly in patients with severe osteoporosis [1]. The bones of osteoporotic patients are structurally weak, often failing to provide adequate fixation for long stems [4]. This mismatch between the stem and the fragile bone can lead to additional fractures, particularly when the femur is anterolaterally bowed [10]. The excessive curvature of the femur restricts the use of long stems, increasing the likelihood of mechanical complications such as stem loosening and further fractures due to the inability of the stem to conform to the natural curvature of the femur [6]. These complications may necessitate further surgical interventions, leading to a significant decrease in the patient’s quality of life [5].

Strut allografts present a viable surgical option for augmenting fragile bone [7]. Dual strut allografts, applied on both sides of the femur, can significantly enhance bone structure strength and provide a promising method for patients with severe osteoporosis [9]. In the current case, we employed long dual strut allografts with a robust cable system. We intentionally crushed the native bone against the loosened femoral stem with sandwiched dual allografts to provide secondary stability to the prosthesis [11]. Although this method may not promote osteointegration between the implant and bone, it enabled the patient to achieve partial weight-bearing capacity and pain-free ambulation, which is particularly beneficial for low-demand patients [12]. An additional advantage of this technique is that it avoids the need for re-approaching the hip joint for stem replacement, which carries the risk of hip joint instability, additional fractures during the dislocation process, and periprosthetic joint infection [2]. By approaching only the femoral shaft, this method may lead to complications related to exposing the hip joint [8].

While partially crushing the native bone to achieve secondary stability showed a good outcome in this case, it should be reserved for selected cases and those with limited indications (Table 1). Given that osteointegration is not expected, this technique may not be suitable for active patients who require full weight-bearing capacity [4]. Additionally, while a cut-off value cannot be suggested, this technique may have limited applicability to patients with a high body mass index. Our patient, who used a cane prior to the injury, was not expected to exert high loads on the fracture site, which influenced our choice of surgical method.

We acknowledge that this is a single case report, and more cases should be studied to validate and refine this approach. Investigating this approach’s applicability across various patient groups and establishing optimized surgical protocols will contribute to setting new standards in orthopedic surgery, ultimately enhancing the quality of life for osteoporotic patients with periprosthetic femoral fracture [11].

## 4. Conclusions

This case suggests that when long-stem revision surgery is challenging in severely osteoporotic patients, partially crushing the native bone with sandwiched dual strut allografts can be an effective alternative [5]. This method provides immediate mechanical stability, promotes early ambulation, and significantly improves outcomes, especially in patients with limited weight-bearing capacity and restricted mobility.

## Figures and Tables

**Table 1:** Suggested indications for using sandwiched strut allograft technique with stem retention in fragile periprosthetic femoral fractures.

Limited weight-bearing capacity/restricted mobility and low body mass index
Osteoporotic fracture with underlying thin femoral cortices
Unable to provide sufficient stability to femoral prosthesis with conventional osteosynthesis surgery
Unable to revise with long femoral stem due to bowing of femur

## Data Availability

All data concerning the case are presented in this manuscript.
